# Antifungal Effects of Terminalia arjuna Fruit Extract on Multidrug-Resistant Candida auris: An In Vitro Study

**DOI:** 10.7759/cureus.107142

**Published:** 2026-04-16

**Authors:** Shailendra K Yadav, Himanshu Dandu, Kishore B Bandamaravuri, Prashant Gupta, Anuradha Nischal, Virendra Atam, Wahid Ali, Ambuj Yadav

**Affiliations:** 1 Department of Medicine, King George's Medical University, Lucknow, IND; 2 Department of Plant Pathology, Council of Scientific and Industrial Research-Central Institute of Medicinal and Aromatic Plants (CSIR-CIMAP), Lucknow, IND; 3 Department of Microbiology, King George's Medical University, Lucknow, IND; 4 Department of Pharmacology, King George's Medical University, Lucknow, IND; 5 Department of Pathology, King George's Medical University, Lucknow, IND

**Keywords:** antifungal, candida auris, candidiasis, drug resistant, terminalia arjuna

## Abstract

Background and introduction: *Candida auris* (*C. auris*) is a multidrug-resistant (MDR) fungal pathogen associated with high global mortality and rapidly increasing infection rates, particularly among immunocompromised patients. This growing resistance highlights the urgent need for new antifungal agents. The bark of *Terminalia arjuna* (*T. arjuna*) is widely used for treating skin ailments such as ringworm (*Tinea* infection) and cutaneous candidiasis. The traditional uses of *T. arjuna* strongly suggest that it may have antifungal activity. Materials and methods: Fresh fruits of *T. arjuna* were collected from the vicinity of Lucknow. The dried fruits (250 g) were subjected to dry distillation to obtain 50 mL of extract, as this method facilitates the recovery of volatile and thermally stable bioactive compounds. The extract was then concentrated to yield 10 mL of a concentrated fraction, which was subsequently dried and used to prepare different concentrations for further analysis. The antifungal efficacy of the extract was tested against clinical isolates of MDR *C. auris*. The antifungal activity of the *T. arjuna* extract was assessed using the disk diffusion susceptibility method for initial screening. Additionally, the minimum inhibitory concentration (MIC) of the extract was determined using a 96-well broth microdilution method. After overnight incubation at 37°C, the diameters of the zones of inhibition were measured and interpreted in accordance with CLSI guidelines. The effect of the extract of *T. arjuna* was compared with fluconazole, amphotericin B, and caspofungin.

Result: The antifungal efficacy of *T. arjuna* extract was evaluated against three MDR clinical isolates of *C. auris* (KGMU-CA-01 to 03) using the disc diffusion method and broth microdilution assay. Baseline resistance profiling confirmed that all isolates were highly resistant (0 mm zone) to both fluconazole (25 ug/disc) and Amphotericin B (20 ug/disc), while the plant extract demonstrated a significant, dose-dependent inhibitory effect. At a concentration of 2000 ug/disc, the extract produced a mean zone of 16.33 mm, which increased to a maximum of 20.33 ± 0.58 mm at 4000 ug/disc in isolate KGMU-CA-03. Statistical analysis via one-way ANOVA confirmed a highly significant dose-response relationship (p = 0.0006), and a comparative analysis showed that the 4000 ug/disc concentration was statistically superior to the active control, caspofungin (5 ug/disc; 15.56 mm average, p = 0.0028). The MIC was 3.12 mg/ml. These comparisons between the crude botanical extract and purified standard drugs were observational and did not imply equivalent pharmacological potency.

Conclusions: The *T. arjuna* fruit extract demonstrated dose-dependent antifungal activity against MDR *C. auris* isolates. The observed inhibitory effects and MIC value indicated promising in vitro antifungal potential of the crude extract. However, these findings were preliminary, based on a crude preparation, and did not imply equivalence to standard antifungal agents. Further studies involving bioassay-guided fractionation and testing on a larger number of isolates were required to validate its therapeutic potential.

## Introduction

*Candida auris* (*C. auris*) has emerged as a significant cause of bloodstream infections in India, particularly in tertiary care settings, where it is associated with high levels of antifungal resistance and considerable mortality. Studies from different regions of India have consistently reported that *C. auris* exhibits high resistance to commonly used antifungal agents, especially fluconazole, with variable resistance to amphotericin B. At the same time, echinocandins remain relatively effective but show emerging resistance [1]. Clinical investigations have also highlighted its role in causing persistent hospital outbreaks and invasive infections such as candidemia, particularly among critically ill and immunocompromised patients [2]. Importantly, these infections are associated with substantial mortality rates, often ranging from approximately 30% to 60%, reflecting the challenges in timely diagnosis and limited therapeutic options [2,3]. The increasing prevalence, MDR, and high mortality associated with *C. auris* underscore its growing public health importance in India and the urgent need for novel and effective antifungal strategies [3].

Systemic fungal infections are an increasing threat in healthcare systems, with *Candida* species being among the most frequent pathogens. One species, in particular, *C. auris*, has gained global attention due to its association with invasive, often fatal infections. First identified in 2009 in Japan, *C. auris* has since caused widespread hospital-associated outbreaks due to its high mortality rate and MDR. Risk factors for infection mirror those for other forms of invasive candidiasis and include recent surgery, central venous catheterization, use of broad-spectrum antibiotics or antifungals, diabetes, neutropenia or immunosuppression, prolonged hospital or ICU stays, and use of indwelling devices like urinary catheters or endotracheal tubes [4,5].

Unlike many other *Candida* species, *C. auris* is frequently misidentified by traditional laboratory methods [6], is notoriously difficult to eradicate from healthcare environments [7,8], and demonstrates resistance to multiple classes of antifungal drugs. Amphotericin B, a polyene antifungal, targets ergosterol in fungal membranes. Resistance arises primarily from mutations in the ERG11 gene, which encodes lanosterol 14α-demethylase, the target enzyme for azole drugs. Mutations such as Y132F and K143R are well-documented and confer cross-resistance across multiple azoles. Approximately 30-40% of *C. auris* isolates are resistant to amphotericin B. Modifications in ergosterol biosynthesis or oxidative stress-related pathways are believed to play a key role. Some *C. auris* isolates show resistance to all three major antifungal classes, azoles, echinocandins, and polyenes, resulting in pan-resistant strains that are extremely difficult to treat and often fatal. Their overall prevalence remains low, with most isolates exhibiting resistance primarily to azoles and variable resistance to amphotericin B, while echinocandins generally remain effective. Such strains have already been identified in the United States, India, and South Africa [9,10]. These traits make *C. auris* especially dangerous in ICUs, long-term care settings, and among immunocompromised patients [11,12].

*Terminalia arjuna* (*T. arjuna*) exhibits notable antifungal activity, attributed to its rich phytochemical profile, which includes tannins, flavonoids, saponins, glycosides, and triterpenoids [13]. These bioactive compounds act through multiple mechanisms to inhibit fungal growth. Tannins denature fungal proteins and disrupt cell wall synthesis, while flavonoids interfere with fungal metabolic enzymes [14]. Saponins and triterpenoids compromise the integrity of fungal cell membranes, leading to leakage of intracellular components and cell death [15]. Various extracts of *T. arjuna*, including aqueous, methanolic, and ethanolic, have demonstrated inhibitory effects against pathogenic fungi such as *Candida albicans*, *Candida tropicalis*, and *Candida glabrata* [16]. Studies using disc diffusion and broth microdilution methods show significant zones of inhibition and low minimum inhibitory concentrations (MICs), suggesting that *T. arjuna* may be a potential natural antifungal agent, particularly in an era of rising resistance to conventional antifungals [17]. The traditional use of *T. arjuna* in wound healing and management of infections suggests the presence of bioactive compounds with antimicrobial properties, which may also extend to antifungal activity. Both bark and fruit extracts have been employed in ethnomedicine, indicating a broad therapeutic potential. These traditional applications, combined with known phytoconstituents such as phenolics and flavonoids with reported antimicrobial effects, provide a plausible rationale for investigating their antifungal activity [18-21].

The primary objective of this study was to evaluate the in vitro antifungal efficacy of *T. arjuna* fruit extract against MDR clinical isolates of *C. auris*. The specific aims were: (i) to characterize the phytochemical profile of the extract using GC-MS; (ii) to determine the susceptibility and zone of inhibition (ZOI) of the extract against three characterized MDR *C. auris* isolates; and (iii) to establish the MIC and compare the extract's inhibitory profile against standard antifungals (fluconazole, amphotericin B, and caspofungin). All comparisons are presented observationally at a standardized concentration (μg/disc), using CLSI interpretive criteria for the control agents to provide a baseline for assessing the extract’s relative potency against characterized resistance patterns.

## Materials and methods

This was an in vitro study. Mature fruits of *T. arjuna* (Roxb. ex DC.) Wight & Arn were collected from Lucknow, Uttar Pradesh, India. Fruits were selected for the present study due to their rich, comparatively underexplored phytochemical profiles. Preliminary evidence suggests that the fruits contain significant amounts of bioactive compounds, including phenolics, flavonoids, and other secondary metabolites, which are known to exhibit antimicrobial activity. The collected fruits were identified and authenticated by experts from the Council of Scientific and Industrial Research - National Botanical Research Institute (CSIR-NBRI), India, and a herbarium voucher specimen (No. PDSH/LWG/Authentication/Ang./2024-25/28) was deposited in the herbarium for future reference. Ethical approval for this study was obtained from our institutional ethics committee (No. 129thECMIIB-Ph.D/P3).

Extraction procedure

The dry (destructive) distillation method was employed for the extraction of the dried fruits of *T. arjuna* [22]. Dry distillation is not a conventional method for phytochemical extraction in antifungal studies. In this study, the technique was employed to isolate volatile, thermally stable bioactive constituents from the fruits. Dry distillation was employed because it does not require a solvent, thereby eliminating potential interference from solvent-related effects. This approach ensured that the observed antifungal activity was attributable solely to the plant-derived constituents, as some solvents used in conventional extraction methods may possess intrinsic antifungal activity and could confound the results. Several compounds identified through chromatography-mass spectrometry (GC-MS) analysis, including phenolics, organic acids, and fatty acid derivatives, were known to remain stable or form active derivatives under controlled thermal conditions. The extraction process was carried out under oxygen-limited conditions with gradual heating, thereby minimizing oxidative degradation and preventing uncontrolled combustion.

Furthermore, thermal decomposition may generate smaller bioactive molecules that contribute to the observed antifungal activity. The mature fruits were shade-dried thoroughly before extraction. Once the extraction apparatus was assembled, 250 grams of dried *T. arjuna* fruits were filled into a 2000 mL borosilicate round-bottom flask. The flask containing the dried fruit material was placed on a heating mantle and connected to a condenser system. The condenser outlet was further connected to two conical collecting flasks maintained under ice-cold conditions to facilitate efficient condensation of vapors. These collecting flasks were connected to an outlet pipe for the controlled release of residual fumes/steam, with the distal end of the pipe immersed in a water-containing vessel to maintain an oxygen-limited environment and minimize the risk of ignition at high temperatures. Carbonization of the dried fruits commenced at temperatures above 270°C, releasing vapors that were directed through the condenser. The temperature was gradually increased up to 400°C under oxygen-restricted conditions. It was observed that exposure to temperatures between 400 and 450°C in oxygen could cause the material to combust; therefore, an oxygen-limited setup was maintained throughout the process. The maximum continuous working temperature of borosilicate glass is approximately 450-500°C, with a recommended safe operating range up to 400-450°C. Heating was discontinued upon completion of the extraction process. A total of 50 ml of extract was collected in a container. The extract was filtered using Whatman filter paper and centrifuged at 5000 rpm. The extract was concentrated to 10 mL using a rotary evaporator, then dried and used to prepare different concentrations for further analysis. The extraction yield was determined as volume per weight (v/w). A total of 50 mL of distillate was obtained from 250 g of dried material and was further concentrated to 10 mL. Upon complete drying to obtain the extract in solid form, 2.3 g of dried extract was obtained, corresponding to a final yield of 0.92% (w/w) relative to the initial dried plant material. The extract obtained was a thermally derived bioactive fraction rather than a conventional solvent-based extract [22].

Strains of MDR-resistant *C. auris*

The microbial strains utilized in this study were procured from the microbial type culture collection at the Advanced Mycology Diagnostic and Research Center (AMDRC), Department of Microbiology, King George's Medical University (KGMU), Lucknow. Specifically, three distinct clinical isolates of *C. auris* (KGMU-CA-01, KGMU-CA-02, and KGMU-CA-03) were selected for antifungal susceptibility testing. These isolates were formally characterized and reported by AMDRC and KGMU as MDR, exhibiting confirmed resistance to both fluconazole and amphotericin B. The use of these characterized clinical strains ensured the study's relevance to current regional resistance patterns.

Antifungal assays

The disc diffusion method was employed as a preliminary/exploratory method used for initial screening of antifungal activity of crude extracts from the dried fruits of *T. arjuna*. Nutrient agar was prepared and aseptically poured into petri dishes. For testing against *Candida* species, Mueller-Hinton Agar supplemented with 2% glucose and methylene blue was used, while 20 mL of Sabouraud Dextrose Agar was used for yeast. The media were inoculated with 100 µL of fungal suspension containing 10⁶ CFU/mL for yeasts and 10⁴ spores/mL for molds. The extract was obtained in dried form and could not be used directly for the assay; therefore, it was dissolved in a suitable solvent. Based on its solubility profile, dimethyl sulfoxide (DMSO) was selected. The extract was subsequently diluted in DMSO to obtain concentrations of 50 mg/mL, 100 mg/mL, and 200 mg/mL, which were chosen for preliminary antifungal screening. For the disk diffusion assay, 20 µL of each concentration was applied per disc, corresponding to 1000 µg/disc (50 mg/mL), 2000 µg/disc (100 mg/mL), and 4000 µg/disc (200 mg/mL). The selected concentration range was primarily based on the solubility of the crude extract in DMSO and its achievable yield following extraction.

Additionally, these concentrations were chosen to enable preliminary screening across a gradient sufficient to observe a dose-response relationship. This approach was consistent with exploratory antifungal studies of crude plant extracts, where relatively higher concentrations were often required due to the presence of both active and non-active constituents. Sterile paper discs (6 mm, HiMedia) were impregnated with 20 µL of each concentration (1000 µg/disc, 2000 µg/disc, and 4000 µg/disc) and placed on the inoculated agar plates under aseptic conditions. Antifungal activities were compared with standard antifungal discs, amphotericin B (20 µg/disc), fluconazole (25 µg/disc), and caspofungin (5 µg/disc), used as positive controls, while 10% DMSO served as the negative control. Given that CLSI/EUCAST interpretive criteria for *C. auris* in disk diffusion are limited, the observed inhibition zones were considered exploratory and used for preliminary comparison only. The MIC determined by the broth microdilution method was regarded as the gold standard for assessing antifungal activity. The inoculated plates were incubated at 37°C for 24 hours, following standard conditions recommended for antifungal susceptibility testing of *Candida* species. Zone diameters were measured after 24 hours using a HiAntibiotic ZoneScale-C ruler and recorded in millimeters. Incubation beyond 24 hours (up to 48 hours) was performed only when required to confirm adequate growth, ensuring consistency and reliability of measurements [23].

Gas (GC-MS) identification

The extract was mixed with 350 µL of cold methanol (-20°C), vortexed for two minutes, and centrifuged at 6000×g for 10 minutes. The supernatant was collected and stored in a glass vial. This procedure was repeated three times, and all supernatants were pooled. After adding 300 µL of chilled water (4°C), the sample was vortexed again and centrifuged under the same conditions, and the resulting supernatant was combined with the methanolic fraction. Ribitol (40 µL of 0.005 mg/mL) was added as an internal standard, and the sample was then dried. For derivatization, 40 µL of methoxyamine hydrochloride dissolved in pyridine (20 mg/mL) was added, and the mixture was incubated at 70°C for 1 hour in an orbital shaker (1000 rpm). Following methoximation, 70 µL of MSTFA + 1% TMCS was added for silylation, and the sample was incubated again at 70°C for one hour. The metabolites were separated on a Trace GOLD TG-5MS column (Thermo Scientific) with dimensions of 30 m length, 0.25 mm internal diameter, and 0.25 µm film thickness. Ultra-high purity helium and argon were used as the carrier and collision gases, respectively, at a flow rate of 1 mL/min [24].

Broth microdilution method (96-well microplate assay)

To determine the MIC of the extract against MDR *C. auris*, the broth microdilution method was performed in a 96-well microplate following CLSI M27-A3 guidelines [25]. The process begins with preparing a stock solution of the extract in 10% DMSO, which is then added to the first column of the plate containing sterile RPMI-1640 medium. A two-fold serial dilution was executed across the plate, usually from columns 1 through 10, to create a decreasing concentration gradient of the compound. Simultaneously, a standardized fungal inoculum was prepared by adjusting a *C. auris* culture to a 0.5 McFarland standard and further diluting it to achieve a final working concentration of approximately 0.5×10³ to 2.5×10³ CFU/mL. This inoculum was then added to all test wells, while specific wells were reserved as positive growth controls (medium plus fungus without extract) and negative sterility controls (medium only). After the plate was sealed to prevent evaporation, it was incubated at 37°C for 24 to 48 hours. The MIC was determined as the lowest concentration of the extract that resulted in complete (100%) inhibition of visible fungal growth compared to the growth control, corresponding to the MIC₁₀₀ endpoint, in accordance with CLSI broth microdilution guidelines.

Statistical analysis

SPSS Statistics version 24.0 (IBM Corp. Released 2016. IBM SPSS Statistics for Windows, Version 24.0. Armonk, NY: IBM Corp.) was used for statistical analysis. The one-way analysis of variance (ANOVA) was used to compare the mean antifungal activity of the crude extracts. Statistical significance was defined as a p-value < 0.05, and results were presented as mean ± SD.

## Results

**Figure 1:**
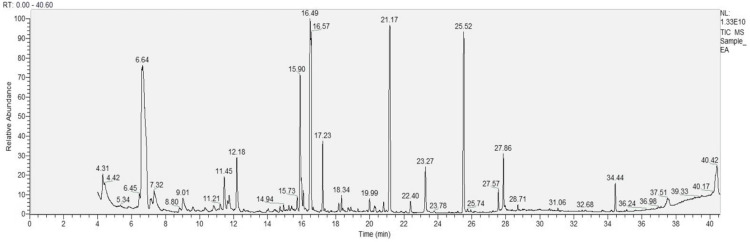
GC-MS profile of T. arjuna fruit extract showing the distribution of bioactive phytochemicals over a 40-minute retention time *T. arjuna*: *Terminalia arjuna*

The GC-MS analysis of *T. arjuna* fruit extract, as shown in Figure 1, revealed diverse metabolites including polyphenols, sugars, fatty acids, organic acids, glycosides, and siloxane derivatives, as listed in Table 1. Some of these compounds have been previously reported to possess antimicrobial properties. Phenolic compounds can disrupt fungal cell walls, inhibit enzymes, and induce oxidative stress, while sugars such as galactopyranoside, glucopyranoside, D-glucose, and D-sorbitol interfere with biofilm formation, nutrient uptake, and osmotic balance. Fatty acids (palmitic and stearic acid) disrupt fungal membranes, and citric acid alters pH and chelates ions, further restricting fungal growth.

**Table 1:** Metabolites identified in T. arjuna extract by GC-MS with corresponding retention times Retention time was expressed in minutes (min). Metabolites were identified by GC-MS analysis using spectral matching against standard libraries. Ribitol was used as an internal standard for normalization. *T. arjuna*: *Terminalia arjuna*, GC-MS: chromatography-mass spectrometry

Retention time (min)	List of metabolites
6.64	Trimethyl phosphate
11.45	2,10-Dioxa-1,12-disiladodecane
12.18	Methyl β-D-galactopyranoside
15.90	Levoglucosan (1,6-anhydro-β-D-glucopyranose)
16.49	Ribitol (internal standard)
17.23	Phosphoric acid
18.34	Citric acid
21.17	D-Sorbitol
22.40	D-Glucose
23.27	n-Hexadecanoic acid (palmitic acid)
25.52	myo-Inositol
27.86	Octadecanoic acid (stearic acid)
34.44	Methyl α-D-glucopyranoside
40.42	Methyl cholate

**Figure 2:**
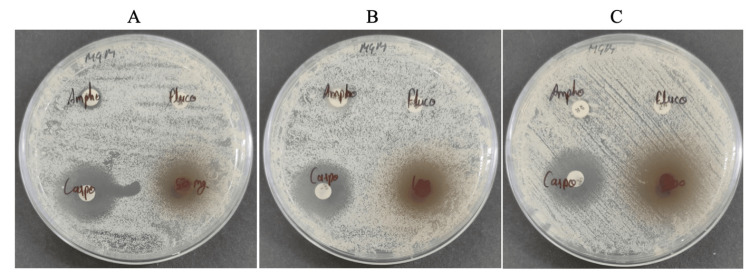
Zone of inhibition of T. arjuna fruits extract against MDR C. auris The extract was diluted in DMSO to obtain concentrations of 50 mg/mL, 100 mg/mL, and 200 mg/mL for preliminary antifungal screening. In the disk diffusion assay, 20 µL of each concentration was applied per disc, corresponding to 1000 µg/disc (A: 50 mg/mL), 2000 µg/disc (B: 100 mg/mL), and 4000 µg/disc (C: 200 mg/mL), respectively. *T. arjuna*: *Terminalia arjuna*, *C. auris*: *Candida auris*, MDR: multidrug-resistant, DMSO: dimethyl sulfoxide

The antifungal efficacy of *T. arjuna* extract against three MDR clinical isolates of *C. auris* (KGMU-CA-01, KGMU-CA-02, and KGMU-CA-03) was summarized in Table 2. The baseline resistance profiling confirmed that all three isolates were highly resistant to the primary antifungal classes, exhibiting a complete absence of a zone of inhibition (0 mm) for both fluconazole (25 ug/disc) and amphotericin B (20 ug/disc). In contrast, the *T. arjuna* extract demonstrated potent, dose-dependent antifungal activity across all tested strains. At the lowest concentration of 1000 ug/disc, the mean zones of inhibition ranged from 13.67 ± 0.58 mm to 14.33 ± 0.58 mm. As the concentration was increased to 4000 ug/disc, the inhibitory effect strengthened significantly, reaching a maximum zone of 20.33 ± 0.58 mm in isolate KGMU-CA-03, as illustrated in Figure 2. The MIC determined by the broth microdilution method (96-well microplate assay) was 3.12 mg/mL. While this value was higher than those of purified standard antifungals, it represented the activity of a crude botanical extract containing a complex mixture of bioactive and non-active compounds. In the context of ethnobotanical research on *C. auris*, an MIC in the low mg/mL range for a crude extract was considered a significant baseline for further bioassay-guided fractionation. This value was therefore presented as a preliminary indicator of antifungal potential rather than as equivalent to clinical-grade pharmaceutical potency.

**Table 2:** Antifungal activity of T. arjuna extracts against MDR C. auris clinical isolates The table presents the mean zones of inhibition (mm) for *T. arjuna* bark extract at three concentrations (1000, 2000, and 4000 µg/disc) compared to standard antifungal agents. Values are expressed as the mean ± SD of triplicate experiments (n = 3). Baseline resistance profiles for clinical isolates (KGMU-CA-01 to 03) indicate high-level resistance to fluconazole and amphotericin B. *T. arjuna*: *Terminalia arjuna*, *C. auris*: *Candida auris*, MDR: multidrug-resistant, FLU: fluconazole, AMB: amphotericin B, KGMU: King George's Medical University, CA: *Candida auris*, SD: standard deviation

*C. auris* strains ID	Resistance profile (baseline)	Zone of inhibition (mm)					
		Extract at different concentrations (µg/disc)			Standard		
					Amphotericin B	Fluconazole	Caspofungin
		1000 µg/disc	2000 µg/disc	4000 µg/disc	20 µg/disc	25 µg/disc	5 µg/disc
KGMU-CA-01	MDR (FLU, AMB)	14.33 ± 0.58	16.33 ± 0.58	19.00 ± 1.00	0	0	15.67 ± 0.58
KGMU-CA-02	MDR (FLU, AMB)	13.67 ± 0.58	15.00 ± 1.00	18.33 ± 0.58	0	0	14.67 ± 0.58
KGMU-CA-03	MDR (FLU, AMB)	14.00 ± 1.00	16.67 ± 0.58	20.33 ± 0.58	0	0	16.33 ± 0.58

The comparative analysis between *T. arjuna* extract and conventional antifungal agents provides preliminary observational insights into its potential as a therapeutic alternative for MDR infections. It is important to note that these comparisons are observational in nature and do not imply equivalent pharmacological potency between the crude botanical extract and the highly purified standard antifungal compounds. The clinical isolates exhibited absolute resistance to the two most commonly used antifungal classes, as defined by established interpretive criteria. Following CLSI M27 guidelines, no zone of inhibition (0 mm) was observed for fluconazole (25 ug/disc), consistent with high-level resistance in *C. auris*, typically mediated by ERG11 mutations or efflux pump overexpression. Similarly, no zone of inhibition (0 mm) was observed for Amphotericin B (20 ug/disc), suggesting significant alterations in ergosterol content of the fungal cell membrane, which is often a hallmark of advanced MDR *C. auris* strains.

Statistical analysis using one-way ANOVA confirmed that the observed dose-response relationship was highly significant (p = 0.0006). Furthermore, observational comparative analysis revealed that the crude extract at 4000 ug/disc (19.22 mm average) produced statistically superior zones of inhibition compared to the active pharmaceutical control, caspofungin (5 ug/disc), which yielded an average zone of 15.56 mm (p = 0.0028).

It was important to qualify that these comparisons were primarily observational, as they involved a crude botanical extract versus a highly purified standard antifungal agent and did not imply equivalent pharmacological potency. However, the consistent inhibitory activity of *T. arjuna*, particularly against isolates that were completely resistant to first-line azoles and polyenes, suggested that it may serve as a valuable source of bioactive leads for the development of alternative therapeutic agents against pan-resistant *C. auris* infections in which conventional treatments have failed.

## Discussion

The present study demonstrates that the extract exhibits concentration-dependent anticandidal activity under in vitro conditions, as evidenced by the progressive increase in the zone of inhibition with increasing extract concentrations. This dose-dependent response suggests that the bioactive constituents present in the extract may exert a cumulative inhibitory effect on fungal growth. Similar concentration-dependent antifungal activity of plant-derived extracts against *Candida* species has been widely reported using agar diffusion and broth microdilution methods [26]. The antifungal efficacy observed in the present study is consistent with earlier reports indicating that *T. arjuna* is rich in diverse phytochemicals, including flavonoids, tannins, and triterpenoids, which contribute to its antimicrobial potential. These compounds are known to disrupt fungal cell membrane integrity, interfere with ergosterol biosynthesis, and induce oxidative stress, ultimately leading to fungal cell death. Previous studies have demonstrated that phytochemical-rich plant extracts exert antifungal effects through mechanisms involving membrane destabilization and enzymatic inhibition. Additionally, phenolic constituents such as catechins, gallic acid, and ellagic acid present in *T. arjuna* have been reported to inhibit fungal biofilm formation and enhance the susceptibility of resistant *Candida* strains [27].

Previous studies supported the present findings; however, greater emphasis was placed on MIC-based evaluations, which are more reliable for antifungal susceptibility testing, particularly for *C. auris*. The extract demonstrated antifungal activity with an MIC of 3.12 mg/mL, indicating measurable inhibition under broth microdilution conditions. Disc diffusion results were interpreted cautiously and used only as supportive evidence. The observed activity may be attributed to mechanisms such as membrane disruption, inhibition of spore germination, and enzymatic interference [27]. Collectively, these results highlight the potential of *T. arjuna* as a promising natural source for developing novel antifungal agents targeting MDR *C. auris*. However, further studies, including in vivo validation, toxicity profiling, and clinical investigations, are necessary to confirm its therapeutic applicability. The MIC determination further substantiates the extract's antifungal potential, demonstrating its ability to inhibit fungal growth at moderate concentrations. Standardization of inoculum density (~1 × 10⁶ CFU/mL), reproducibility across triplicate experiments, and statistically significant differences (p < 0.05) strengthen the reliability and validity of the findings. These observations align with established antifungal susceptibility testing protocols and previous validation studies [28].

Importantly, when compared with conventional antifungal agents, the *T. arjuna* extract demonstrated comparable or superior efficacy against the MDR *C. auris* isolate. The lack of significant activity observed with fluconazole and amphotericin B confirms the strain's resistant phenotype. In contrast, the relatively lower inhibition observed with Caspofungin highlights the potential advantage of the plant extract. Similar findings have been reported where plant extracts enhanced antifungal activity against resistant *Candida* strains. Furthermore, the enhanced antifungal activity observed at higher extract concentrations may suggest interactions among phytoconstituents that warrant further investigation. Previous studies have reported synergistic interactions between plant-derived compounds and antifungal agents, resulting in increased antifungal potency and reduced resistance [29]. These interactions may involve increased membrane permeability, inhibition of efflux pumps, or disruption of fungal metabolic pathways.

Limitations

The study is primarily limited by the use of a crude extract and a dry distillation process, which may cause thermal degradation of heat-sensitive bioactive compounds. Additionally, the in vitro nature of the assays does not account for host metabolism or bioavailability, and the diffusion-dependent disc diffusion method may vary based on compound solubility. Finally, the absence of established clinical breakpoints for botanical extracts means that all comparisons to standard antifungals remain purely observational. The experiments were conducted only under laboratory conditions against *C. auris*, which may not fully reflect the complex physiological conditions of living systems; therefore, the efficacy of the *T. arjuna* fruit extract may vary in vivo. Additionally, the study included a limited number of clinical isolates from a single center, which may restrict the generalizability of the findings. Although GC-MS analysis identified several phytochemicals, the specific active compounds responsible for the antifungal activity were not isolated, and toxicity or safety evaluation was not performed. Therefore, further studies involving purification of active constituents, toxicity assessment, mechanistic investigations, and in vivo validation are required to confirm the therapeutic potential of *T. arjuna* against MDR *C. auris*.

## Conclusions

The present in vitro investigation demonstrated that the fruit extract of *T. arjuna* exhibited antifungal activity against MDR *C. auris*. The extract showed a clear concentration-dependent inhibitory effect and demonstrated greater inhibitory activity than the active control, caspofungin, under the tested conditions. Notably, the extract remained effective against the tested isolates, whereas fluconazole and amphotericin B exhibited no detectable inhibitory activity, confirming the strains' MDR. The MIC, determined by broth microdilution assay, further supported the extract's antifungal potential. Chemical profiling by GC-MS revealed the presence of multiple bioactive metabolites, including phenolic compounds, fatty acids, organic acids, sugars, and glycosides, which have been reported to possess antifungal properties through mechanisms such as membrane disruption, oxidative stress induction, enzyme inhibition, and interference with biofilm formation. These phytochemicals likely contributed to the extract's observed antifungal activity. Overall, the findings highlighted *T. arjuna* fruit extract as a promising natural antifungal candidate against MDR *C. auris*. However, these findings were preliminary, based on a crude extract, and did not imply equivalence to standard antifungal agents. Further studies involving bioassay-guided fractionation, testing on a larger number of isolates, mechanistic evaluation, toxicity assessment, and in vivo validation were required to establish its therapeutic potential and clinical applicability.
